# Five years on: analysis of university lecturers’ experiences of the French government’s health promotion education program

**DOI:** 10.1186/s12909-024-05755-x

**Published:** 2024-08-07

**Authors:** Mathilde Monpierre, Adèle Frachon, Alexandra Eguiluz, Pauline Martinot, Annabelle Tenenbaum

**Affiliations:** 1 Educations and Health Promotion Laboratory UR 3412, UFR Health, Medicine, Human Biology, Paris Sorbonne Paris Nord University, Bobigny, 93000 France; 2 Service d’Accueil des Urgences - SAMU - SMUR, CHU de la Guadeloupe, Pointe à Pitre, 97159 Guadeloupe France; 3 Université des Antilles, Pointe à Pitre, 97157 Guadeloupe France; 4 Département de Médecine Générale, Université Paris Cité, Paris, 75006 France; 5 Université Paris Cité, Paris, 75006 France; 6 Learning Planet Institute, Paris, 75004 France; 7 Cognitive Neuroimaging Unit, NeuroSpin Center, CEA, INSERM, Paris-Saclay University, Gif/Yvette, 91191 France; 8 College of France, Paris-Sciences-Lettres University, Paris, 75231 France; 9 UFR d’Odontologie, Université Paris Cité, Paris, 75006 France; 10 Department of Oral and Dental Medicine, APHP, Groupe Hospitalier Pitié Salpêtrière, Paris, 75013 France

**Keywords:** University lecturers’ experiences, Health students, Prevention, Health promotion, Education, Health curriculum

## Abstract

**Background:**

The National Health Promotion Intervention Program by Student (HPIPS) is a French government educational program introduced in 2018, aiming at developing all health students’ health promotion knowledge and abilities, as well as implementing health promotion interventions for specific subpopulations in the general public. Its pedagogical framework was elaborated in 2018 and then evaluated by the French Council for Public Health in 2022, highlighting certain difficulties for the program to be homogeneously implemented in France. The aim of this study was to explore and describe the experiences and feedback of university lecturers in charge of this HPIPS training.

**Methods:**

Semi-structured interviews were conducted with HPIPS lecturers from various health fields and from French universities, and a qualitative content analysis was carried out.

**Results:**

Fourteen interviews were conducted during the autumn of 2022 with HPIPS program university lecturers including five doctors, three dentists, two nurses, two pharmacists, one midwife, and one physiotherapist from eight different towns belonging to six regions. Depending on the professional background, the component, and the local resources available, the teaching experience varied from one lecturer to another. A number of difficulties arose in setting up this educational program and complying with the latter legislation. The work overload was considerable, and the lecturers’ heavy commitments some lecturers to be discouraged, especially since some lecturers were not trained in health promotion abilities. Although interprofessionality was a strength of this HPIPS, it was also its main challenge. Pedagogical innovations were developed, notably through the use of digital technology; cross-disciplinary collaboration was established; and lecturers–students specific boundaries have emerged thanks to this health promotion project.

**Conclusions:**

In France, setting up the HPIPS rapidly was experienced as a real pedagogical challenge for the interviewed university lecturers. While most of them noted the positive and beneficial contributions made by the introduction of prevention and health promotion intervention skills for health students, they also shared recommendations in order to match the ambitions and increase the HPIPS impact on the development of a culture of prevention and health promotion among health students.

## Background

The National Health Promotion Intervention Program by Students (HPIPS) is an annual French national program targeting all health students, including those in medicine, dentistry, midwifery, nursing, physiotherapy and pharmacy (French name ‘Service Sanitaire des Etudiants en Santé’). The program had two main objectives: (1) to train health students in health promotion interventions and (2) to implement these interventions for specific subpopulations within the general public. The HPIPS was launched in the academic year 2018 (starting in September in France) at the request of the French government. The short time left for universities to plan the HPIPS in September 2018 (i.e., official announcement in june 2018) led to the absence of national resources or additional lecturers provided for its implementation. Four training aims were announced as the HPIPS implementation success indicators: “(1) initiating students to the challenges of primary prevention, (2) enabling them to carry out concrete primary prevention interventions, (3) promoting interprofessionality and interdisciplinarity during training courses and interventions carried out, and (4) integrating prevention and health promotion into the practices of healthcare professionals” [1, 2]. Students had a total duration of 6 weeks full-time to be trained for their health promotion interventions, to acquire the needed pedagogical knowledge and skills, to define their targeted population and their thematic, to build on their health promotion intervention under supervision and to plan their evaluation. The HPIPS students training comprised several teaching units including both theoretical and practical training time, such as introduction to prevention concepts in all environments and throughout life; enabling and reinforcing the training of future healthcare professionals in health prevention and promotion; reinforcing their awareness of prevention and health promotion challenges by ensuring their mastery of the necessary knowledge and skills [1]; developing new skills to meet the public health challenges of promoting health-promoting behaviors; contributing to the reduction of social and territorial inequalities in health. This approach to teaching health promotion can be found among students in public health training programs [3], but what makes HPIPS special is that it concerns future healthcare professionals in a multi-professional perspective.

**Table 1:**
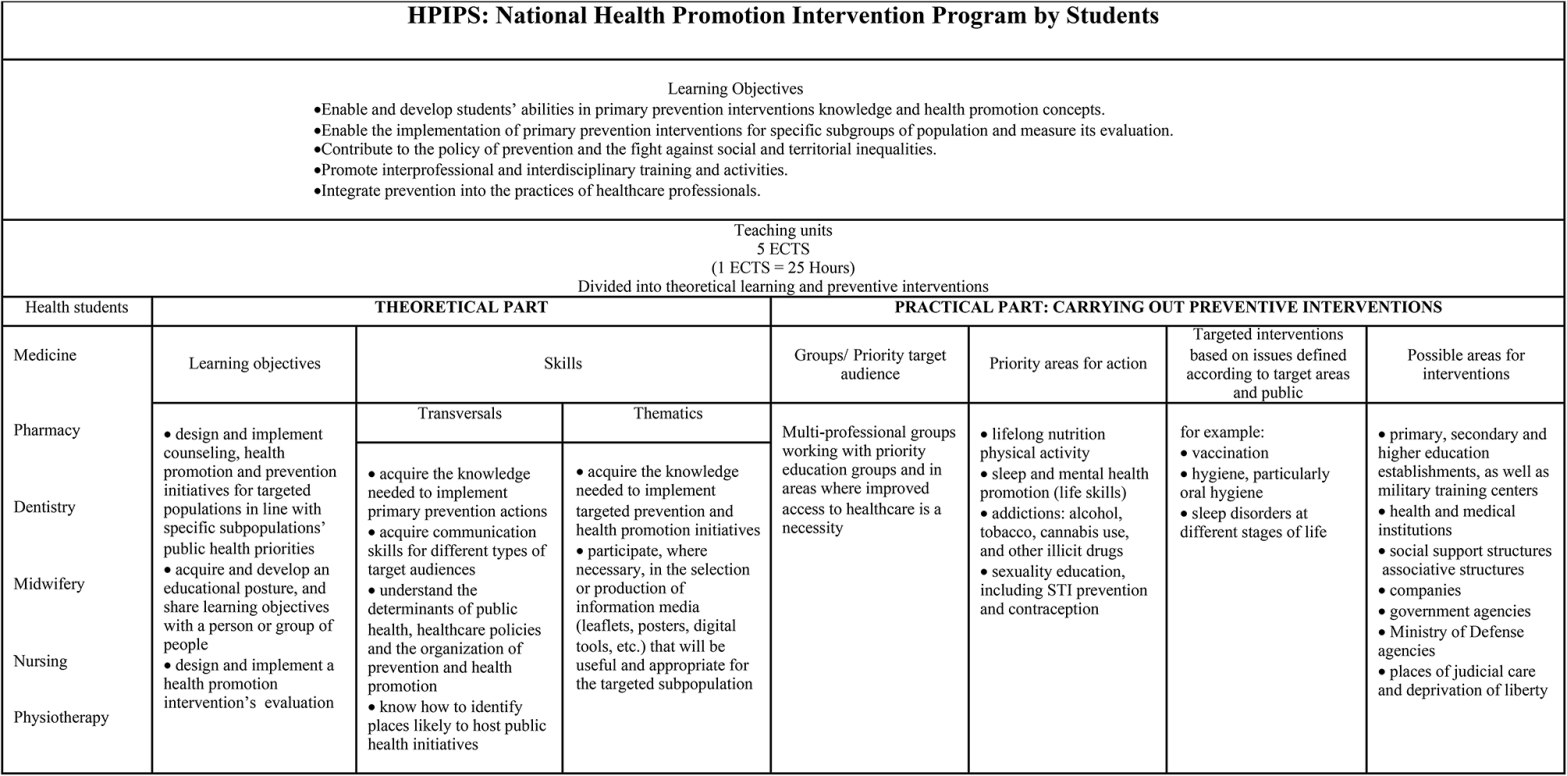
Description of the HPIPS according to decree n°2018 − 472 of June 12, 2018 [2], as amended in 2020

The HPIPS pedagogical framework was elaborated by the Haut Conseil de Santé Publique (HCSP; High council of public health) in 2018 and then, evaluated in 2022 [4]. A number of pedagogical difficulties were identified: the existence of interregional disparities in the organization and pedagogical offers in each field among the 13 regions of France (i.e., in most universities, only few lecturers had enough expertise in the fields of health prevention–promotion training or health communication, an essential prerequisite for the development of health preventive interventions) as well as the presence of a body of documentation described as “too abundant and insufficiently adapted to the particularities of the HPIPS.” One of the report’s recommendations was to “strengthen the capacity of teams to train students in health promotion interventions and health communicative methods, drawing on the resources and know-how of prevention-health promotion practitioners and/or university teams in the humanities or educational sciences in the regions.”

With the aim of sharing experience and improving health promotion intervention teaching program, this study explored and described university lecturers’ experiences, their feedback on the struggles and strengths met during their health promotion teaching within the HPIPS framework. In particular, the study wished to provide useful tips on the implementation of these pedagogical courses and thus leading to an improved training program for health students in health promotion interventions in France.

## Methods

### Approach

The choice of semi-structured interviews allowed us to explore in depth, contextualize and understand the experiences of the lecturers involved. This qualitative method was the most relevant to our research objectives as we wanted to understand lecturers’ experience, feedback and solutions when implementing the HPIPS [5, 6].

### Participants

Participants were recruited through “purposeful sampling”, a method which selected individuals based on specific criteria (described below), relevant to the research objective. This method allowed researchers to collect comprehensive and representative data from the population of interest [7]. The selection criteria included: (1) Being a HPIPS lecturer in a healthcare field (medicine, dentistry, midwifery, nursing, physiotherapy, and pharmacy). (2) Affiliation with different universities. (3) Representation from various regions and cities.

Network sampling was carried out throughout France territory. Recruitment was initially carried out among known contacts representative of the various health components (medicine, dentistry, nursing, pharmacy, midwifery, and physiotherapy) of the professional circle of the various project researchers. Secondly, a “snowball” method [8] was used to extend the survey from person to person. Each participant received an email inviting them to take part in the study, with an information note attached.

### Data collection

Data were collected through in-depth, semi-structured interviews conducted between January 28, 2022, and April 21, 2022. Each participant participated in a one-on-one, face-to-face interview with the researcher. To understand the experiences of HPIPS lecturers, a semi-structured interview guide was developed by several researchers (MM, AE, and AT). The interview questions were formulated by incorporating insights from relevant literature and items identified in the report by the French High Council for Public Health. The main questions in the interview guide were as follows: A/ What do you think of the way HPIPS teaching is organized at your university? How do you feel about it? B) How did you experience the implementation of the HPIPS? What are your expectations and needs for the future? C) How would you describe what the HPIPS has brought to you, the teaching team and the students? D) What would be the ideal HPIPS teaching framework to meet HPIPS objectives (without taking budget constraints into account)?

The interviews were conducted remotely via videoconferencing software (University Zoom License) by one of the researchers (MM). The conclusion of the interview happened once the thematic saturation was reached, i.e. when there were no longer any relevant codes or categories, and recurring topics cease to provide additional insight to the study [9].

### Analysis

The data were analyzed using inductive content analyses, a qualitative method where data are collected until they can be categorized into multiple cases of the same type. This process results in categories based on units of meaning that were consistent, recurrent, and regularly observed [10]. The interviews were recorded with participants’ consent, were anonymized and were transcribed into verbatim. Two dimensions of the analysis were performed: firstly, a vertical one (intra-interview) and secondly, an horizontal one (inter-interview: comparative step between the interviews) [11]. Subsequently, each researcher thoroughly read the transcriptions to gain a comprehensive understanding of each interview. Following this, utilizing the verbatim, the researchers proceeded with open coding, which involved identifying significant recurrent phrases and keywords. The verbatim were analyzed using an Excel Spreadsheet. At this stage, researchers pooled the themes identified to propose categories. A horizontal thematic analysis of each interview was carried out. All interviews were coded by one of the researchers (MM) and co-coded by the other two researchers (AF and AT) in order to triangulate the data (i.e., using multiple perspectives to gain a more comprehensive understanding of a research problem).

### Ethics

A particular care was taken to ensure compliance with ethical standards throughout the research process. An information note containing all the essential information (research framework, objective, right of refusal, right of withdrawal without justification, right of access to the overall results, as well as the researchers’ contact details) was sent by email to the participants. Secondly, after acceptance of participation in the study, both a consent form for participation in the study and a consent form for the audio recording of the interview were signed. The interview was conducted by videoconference in a confidential environment. Information was stored securely and temporarily. After transcription, all interviews were anonymized. A declaration was made to Paris Cité University’s data protection officer.

The study protocol was evaluated by the University of Paris Research Ethics Committee (CER U-Paris). After two sets of recommendations, followed by modifications, the study protocol was approved. The following IRB number was assigned to the project: 00012022-10.

## Results

A total of 14 individual interviews were conducted with five doctors, three dentists, two nurses, two pharmacists, one midwife, and one physiotherapist. The average duration of the interviews was 40 min (ranging from 20 to 60 min). Participants were lecturers that worked in eight different cities located in six different regions. Through their functions, they were able to testify about implementation of the HPIPS in their departments and universities. The characteristics of the lecturers interviewed are provided in Table 2.

**Table 2:** Lecturers’ fields and locations

Identification	Sector	Region-City
**E1**	Nursing	Pays de la loire-1
**E2**	Nursing	Ile de France -1
**E3**	Midwife	Ile de France -2
**E4**	Medicine	Ile de France -2
**E5**	Medicine	Pays de la Loire -1
**E6**	Pharmacy	Occitanie -1
**E7**	Medicine	Grand-Est-1
**E8**	Medicine	Nouvelle-Aquitaine -1
**E9**	Medicine	Ile de France -2
**E10**	Dentistry	Haut-de-France -1
**E11**	Dentistry	Nouvelle-Aquitaine -1
**E12**	Dentistry	Grand-Est -2
**E13**	Pharmacy	Nouvelle-Aquitaine- 1
**E14**	Physiotherapy	Grand-Est -2

**Fig. 1:**
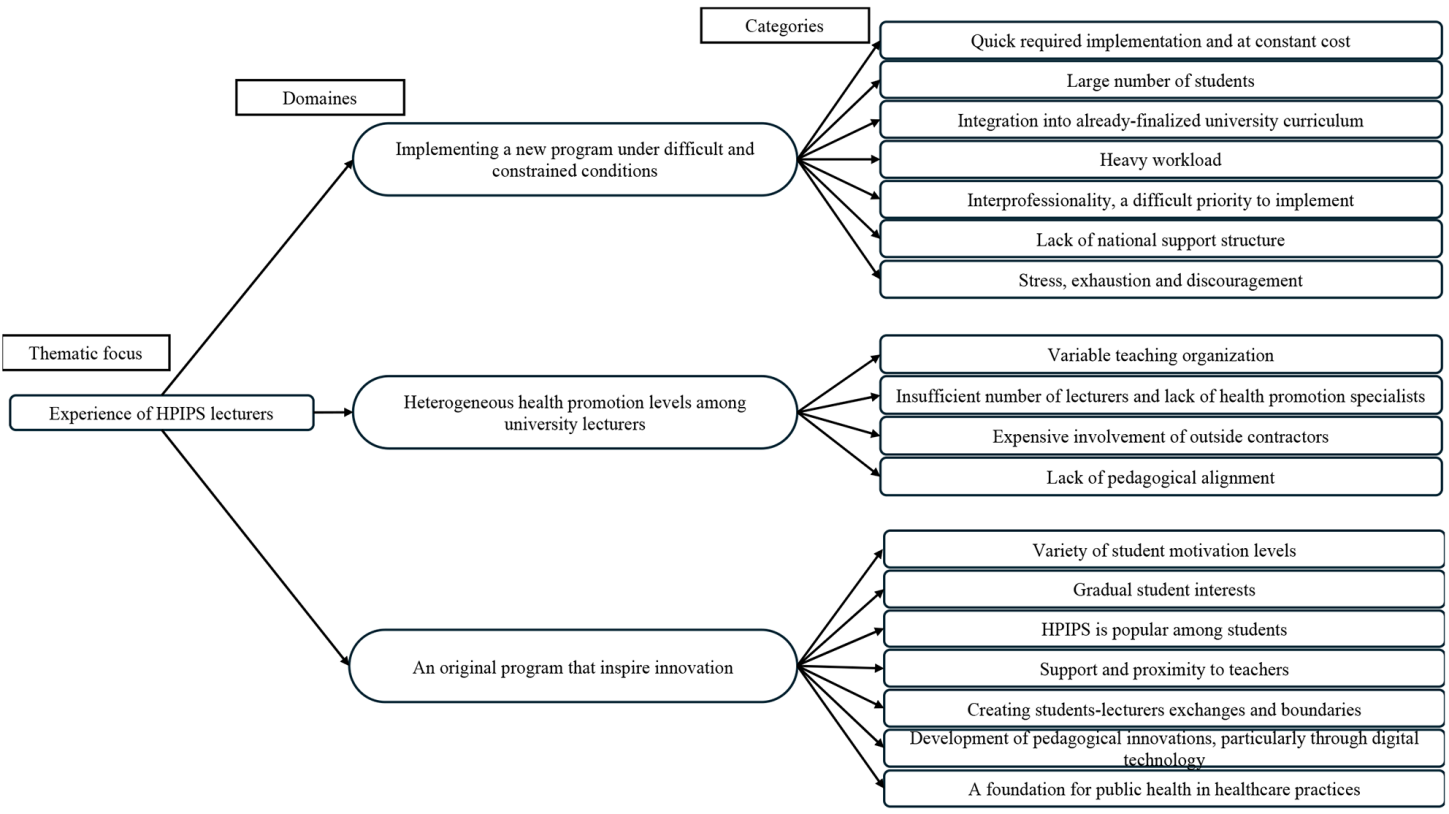
Theme tree

Three domaines – (1) Implementing a new program under difficult and constrained conditions; (2) Heterogeneous training levels among lecturers in health promotion; and (3) an original program that inspired innovations, as well as 18 theme categories emerged from the qualitative analysis (Fig. 1).

### Implementing a new program under difficult and constrained conditions

#### The facts

Regarding difficulties and encountered constraints, lecturers particularly regretted the fact that they had to implement the HPIPS very quickly (i.e., in 2 months, during the summertime break, on year 2018), without any health promotion official resources; additionally, they had to addressed the program for a large number of students (i.e., 47,000 students were concerned per year) with a low number of university lecturers, within teaching curriculum that had already been finalized, all of which generated a heavy workload: “We had application decrees that came out in July, and our management teams wanted them to be effectively implemented from the start of the new school year in September” (E2); “The HPIPS is an additional teaching load that has been added on at constant cost” (E7). Interprofessionality was an additional priority to consider within the HPIPS. On the one hand, for some lecturers, developing interprofesionnality within health students was positively welcomed: “I really wanted to enable interprofessionalism, to prioritize it” (E14); “It also means learning to work with each other, to know each other’s areas of expertise…” (E2). On the other hand, although it was seen as essential, the implementation of interprofessionality was judged to be very complicated given the reality on the ground: “It’s really very complicated to coordinate all that” (E14). Making six health curricula match for a 6-weeks full time work appeared hard to implement. Some lecturers also pointed disparities between courses, with “students at very, very different levels” (E10), leading to teaching objectives that were hard to articulate consistently. Furthermore, if multiprofessional training was to be achieved within the same timeframe, matching curricula is a prerequisite and requires a great deal of upstream anticipation, which was not the case with the hasty introduction of the HPIPS.

In addition to being implemented in the teaching curriculum, it was extremely time-consuming to find health promotion interventions’ sites for all the students, with up to “1,200 actions for some components” (E8). The lecturers reported a high level of investment in meeting the HPIPS ambitions, with few resources allocated. In the face of the many difficulties encountered, lecturers witnessed a high level of stress, exhaustion, and discouragement: “We put a lot, a lot of energy into setting up this health service with constant resources” (E10); “It was a major stress factor…” (E7); “That’s it, I’ve given up” (E10).

Furthermore, lecturers reported a lack of national guidance to oversight, create boundaries between universities’ programs and centralize training resources. “That’s one of my regrets: we don’t have any feedback from other faculties… outside my own town, I don’t have a vision of the health service in other faculties” (E6). In addition, it is important to note that HPIPS’ assessment methods varied widely across the territory. Assessments were allowed to be multimodal (oral, written, presence), unimodal, or were absent for some university.

### Expectations

Lecturers reported three main areas of improvement for the HPIPS: (1) The provision of resources: additional dedicated time and financial and human resources (e.g., lecturers, tutors) and support from local and regional authorities. (2) A provided support for the organization of teaching and activities, enabling the goal of interprofessional collaboration to be achieved, based on a common, structured HPIPS over and above the various disciplines. (3) A curricular approach enabling the program to be spread over several semesters, with a vision of progression in three training cycles: “Let the first cycle be the HPIPS; it’s a first approach where they are mere observers after all… In the second cycle, integrate into clinical practice… And then in the 3rd cycle, implement coordinated therapeutic strategies” (E8). Such an overhaul would give lecturers back their autonomy and ease the pressure on teaching models.

### Heterogeneous training levels among lecturers in Health Promotion

#### The facts

Lecturers pointed to a variable quality of organization of teaching, an insufficient number of lecturers, and a lack of field workers trained in health promotion: “We call on colleagues who don’t have this public health culture and who think in the same way as the students” (E10). To make up for this shortage of lecturers trained in health promotion, the lecturers in charge of the HPIPS have mobilized a large number of outside contributors to help design the training courses, run the tutorials, and monitor the groups’ interventions. Training courses offered by organizations specializing in health education and promotion, such as the Regional Institutes for Health Education and Promotion (IREPS) or the Departmental Health Education Committee (CODES) for HPIPS supervisors have been extremely useful. However, these training courses were dependent on agreements between the authorities and the university, were annually signed, and only took place occasionally during the year. In some cases, collaborations with external partners were restrained as no defined budget was defined for these services.

Moreover, there was a desire for the various components to co-construct the courses, with a specific amount of time devoted to this task and particular attention paid to ensuring that the program is not overloaded. Some lecturers also mentioned the need for specific hybrid HPIPS training—“Something dematerialized, simple, not too heavy” (E5)—in initial training, available to lecturers, and upgradeable: “I think it would be necessary that every year, there is an update of knowledge, if only on the tools” (E1).

One lecturer expressed his opinion on what he considered to be a discrepancy in the HPIPS between the objective of the initial training of students (i.e., awareness level) and health education actor’s missions (i.e., in-depth level). This lecturer stated that a health promotion intervention requires specific, highly advanced skills that cannot be achieved in such a short training program for students with little maturity and understanding of the field of public health: “Intervention in health promotion requires skills and experience. These are the main quality criteria. Normally, it requires at least a type 2 master’s degree and a very good knowledge of the public concerned” (E8). To achieve such knowledge, he argued, the HPIPS should focus more on training students; it should be a pedagogical device aimed strictly at theoretical learning and should not go as far as intervention: “…intervention doesn’t make sense, they’re too young, they don’t know how to do it” (E8).

### Expectations

The lecturers mentioned two main areas for pedagogical improvement: (1) to obtain shared pedagogical resources on a regional scale (E5: “Try at least on a regional scale, to harmonize the lecturers we have done remotely”) and/or nationally (E13: “I would have liked to see a national platform with certain points…materials that would be short, engaging for students with key and practical points”); and (2) to open additional teaching positions and pedagogical referents (E5: “It’s a project that’s fairly circumscribed around a few lecturers, which will perhaps benefit from being extended a little, bringing people into the health service adventure”) by “creating positions” (E9) for this purpose.

### An original program that inspires innovation

#### The facts

Lecturers reported a gradual increase in interest in the HPIPS on the part of students. At first, they were perceived by the lecturers as reluctant to implement the HPIPS: “We had a lot of resistance at the start” (E1). Some students did not attend class and had little interest in the theoretical online lessons. The level of motivation varied: “There are students who will be interested, who will be highly motivated, who will follow it well. And then there are other students who will do it just to pass the exam” (E11); “I think it’s an exercise that students really appreciate” (E8). Despite the perceived unmotivated students, it seems that the HPIPS was highly appreciated by students, especially the project-based and interprofessional work: “I think students really appreciate this collaborative work, getting to know each other better, discussing, exchanging and working together” (E2).

The lecturers believed that the introduction of the HPIPS had been enriching for the students— “I can see the shift from curative to preventive care… And that’s when we say to ourselves, well, this teaching is useful” (E9)—and also for the lecturers: “I’ve learned a lot thanks to the HPIPS” (E4).

The HPIPS was seen as an opportunity to develop a gesture of support and proximity to students—in other words, to create boundaries outside the usual university framework: “It allows us to create a more important exchange to support them on a project… We really have a posture of support” (E2).

Furthermore, for lecturers, setting up the HPIPS has enabled them to develop pedagogical innovations, particularly through digital technology: “We really created a studio with a technician who filmed different shots, and tools that were specially created for this purpose” (E7).

There was a need for innovation (E1: “It also forced us to get moving to hybridize…”) and teamwork in the form of collaboration with other departments and partners (E10: “It was very enriching because it led us, from a human and relational point of view, to work with our colleagues from other components…something we’d never done before”).

The result of such collaboration was the creation of exchanges and links: “It has enabled us to create something we had very little of: links between administrators and lecturers” (E5). Moreover, the HPIPS created opportunities by breaking away from the usual framework of internship sites: “It has also enabled them [the students] to break away from a very hospital-based or clinical approach to internships” (E1). For some lecturers, the formalization of such a program, with a decree very clearly defining the course to be followed with competency objectives and an obligation to implement it in all courses, had led to administrative recognition: “I think that for the administration…there is recognition; the public health laboratory has had greater identification on the faculty” (E6).

### Expectations

Lecturers noted two main areas for improvement: (1) Encouraging pedagogical innovation (E2: “I think we need to innovate. Pedagogical innovation is very important; we need to adapt to today’s students”) and sharing resources (E5: “I think we’re going to do better and at a lower cost”). (2) Considering how to organize and collaborate to reduce social and territorial inequalities in health, and considering moving away from metropolises: “Go to the towns with the lowest density of doctors, the least access to health care, and so really break out into the territory with a targeted approach” (E5).

## Discussion

The aim of this study was to explore and describe university lecturers’experiences of the national prevention and health promotion program offered as part of the HPIPS in France and launched in 2018. The choice of conducting a qualitative study was justified by the need to apprehend the experience of differents actors and by collating a diversity of viewpoints. Experiences vary from one lecturer to another depending on professional background and the available local resources.

It is only since the 1990s that health promotion and health education have gradually become established in the French healthcare system during the initial training of healthcare professionals [12]. Since 2018, the national strategy to establish a “policy of health promotion in all living environments” [13] has strengthened the place of health promotion in the French healthcare system. The HPIPS was one of the measures taken under this policy [2]; it was France’s first major national educational program in health promotion for future healthcare professionals. HPIPS lecturers found themselves on the front lines, with a complete educational program to plan and deploy, taking into account administrative requirements, field constraints, and available resources. Implementing the program with a continuous supply of resources led to the development of pedagogical innovations [14], with varying needs for pedagogical reengineering [15] as well as for the development of digital technology, which is booming in the field of health science pedagogy [16]. The use of sometimes costly external contributors has often been necessary due to a lack of local resources, thus raising the question of the sustainability of training schemes.

Professional relations have also evolved with the introduction of the HPIPS. The latter has demanded the birth of exchanges and new boundaries between lecturers and/or cross-faculty referents to establish interdisciplinarity and the pooling of teaching. The lecturer–student relationship has gradually changed, with the lecturer taking on an accompanying role in the development of the project, with particular involvement at every stage. This relationship combined greater involvement and proximity, with the shared objective of carrying out an action with a third-party audience. The resulting triangulation required the lecturer–student pair to reinvent themselves, as they were seen as a unit by the target population.

However, this training scheme raised a number of difficulties. First, a significant weight of this program on the university curriculum (five European Credit Transfer and Accumulation System, ECTS), which had to be integrated into the current teaching curriculum. Second, the effort involved in reorganizing teaching and implementing this new system resulted in a considerable additional workload for lecturers, which generated stress, exhaustion, discouragement and, was highly dependent on the specific contexts of each component and university. A qualitative case study carried out in two academies in the Nouvelle-Aquitaine region, Poitiers and Bordeaux, confirmed these difficult and uneven implementation conditions, with “rushed implementation, a stricking lack of support and resources…an overload of work” [17]. Third, the results of our study showed that lecturers focused more on the technical conditions for implementing the program than on the pedagogical content to be taught.

While one of the main training aims was to promote interprofessional and interdisciplinary skills, the reality in the field did not fully support this expectation. Lecturers reported numerous organizational constraints, resulting in difficult, if not impossible, to apply it, even though it was a key element of the HPIPS. These constraints included the large number of students, different teaching non-defined formats, non-overlapping timetables, and different stages of student training in public health topics. Moreover, a recent qualitative study was carried out among midwifery school lecturers and directors with the aim of providing feedback on the introduction of the HPIPS within the framework of maieutic studies. Similarly, the study reported “organizational difficulties,” “major differences in reengineering,” and a vision of interprofessionality as “the difficulty and the strength” of the HPIPS [15].

We have noted a heterogeneity in assessment methods. This raises the question of the pedagogical alignment [18] to be established within the HPIPS framework. Pedagogical alignment was defined as “coherence between learning objectives, pedagogical methods and assessment principles and tools” [19]. Assessment strategies varied and were sometimes even nonexistent, while pedagogical activities followed the same goal at the national scale. However, unlike training objectives, learning objectives were not clearly enough defined. In this context, pedagogical alignment was difficult to achieve, which led to the need of core competencies to be defined within the HPIPS, to improve the training of future health professionals in the fields of prevention and health promotion.

Now that a few years have passed since the HPIPS was set up, there were many expectations for improving the system, such as establishing or reinforcing interprofessional collaboration or pooling resources on a regional and/or national scale. A number of projects in this vein were currently underway, with a view to the positive development of the HPIPS. Major investments will be required to counteract the constraints of reality on the ground. For example, in order to strengthen and optimize interprofessionality, “the decompartmentalization of medical and paramedical disciplines requires a complete reorganization of schedules (internships, courses, examinations, …) to ensure that training, group work and joint interventions in host structures coincide” [14].

The HPIPS was viewed by lecturers as an introduction to health promotion for future healthcare professionals, as it is a short-timed, one-off educational scheme. In the HCSP report [4] as well as in two exploratory qualitative studies, the “pedagogical objectives are over-ambitious, referring to levels of training higher than those achievable under HPIPS conditions.” The results of the aforementioned study carried out in two academies in the Nouvelle-Aquitaine region [17] on the objectives of the HPIPS and the interventions carried out by the students call such objectives and interventions into question: “the objectives are revealed to be out of step with the challenges of prevention in the healthcare system; students have endorsed a mistaken vision of health promotion as rational and individualizing health-related behaviors, and the interventions carried out contravene for the majority the quality criteria in health promotion, be they pedagogical, methodological or ethical.” Strengthening the place of health promotion in the French healthcare system must involve improving and reinforcing the training of future professionals in this field. The acquisition of health promotion skills should be envisaged throughout initial training, and it would require a prerequisite inquiry into students’ levels of literacy. Health literacy is defined as “the ability to access, understand, evaluate and communicate information as a means of promoting, maintaining and improving health” [20, 21]. In a quasi-experimental study carried out in a Paris medical school with medical students who had carried out preventive interventions as part of their health service [22], two important results were highlighted: “two thirds of students did not feel sufficiently prepared to carry out preventive health interventions, and for students, reporting a satisfactory experience in health service was associated with reporting the acquisition of skills or knowledge.”

Reforming the HPIPS system by improving health promotion training for future healthcare professionals—for example, by establishing a curricular program with early initiation, continuity of learning over time, and a progression of skill levels according to teaching cycles—might be one way forward [23]. Only if healthcare professionals are properly trained in health promotion and if quality interventions are specifically evaluated, then we could reach clearer results, both in regard to the involvement of these lecturers in health promotion and preventive interventions, as well as among the health students abilities development.

To our knowledge, this work is the first multiprofessional qualitative study of the experiences and feedback of HPIPS French referent lecturers. Strengths of this study covered its recruitement, with cross-sectional (i.e., six health university domain) and cross-regional perspectives. This qualitative study has been designed and realized with rigor, fulfilling the criteria of scientificity in qualitative research [5, 19, 24]: it has shown credibility through the triangulation of data and co-coding; its transferability and reliability within the triangulation of researchers; the independence of the main researcher, not involved in the HPIPS.

The study limitations included the use of videoconference when interviewing lecturers, in view of the health constraints imposed by the COVID-19 pandemic at the time the study was carried out. This modality favored the geographic diversity of participants, allowing flexibility and removing spatial barriers, but might have led to a certain loss of information in exchanges regarding how nonverbal communication was perceived [25]. In addition, the interview guide was long, as it was designed to arouse lecturers’ discourse.

## Conclusion

Lecturers’ feedback and experience of health promotion teaching in the HPIPS varied, depending on professional health promotion and intervention background, pedagogical innovating skills, numerical familiarity, health promotion local outside contributors availabities to support and co-teach the students, the presence of a university public health research unit specialized in prevention and health promotion, as well as other factors. With only two months to prepare their courses in 2018, lecturers have had to develop innovative pedagogical tools to meet the expectations of the HPIPS, notably through digital technology; cross-disciplinary collaborations have been set up. Overall, lecturers’ opinions of the HPIPS were positive, and lecturer’s interviews enabled to identify a need for common cross-functional tools, a defined skills base, and shared resources for health promotion intervention teaching ambitious programs. Five years after the scheme’s application, HPIPS lecturers tended to see the program as a way of raising awareness in prevention and health promotion among future health professionals (i.e., health students), and wonder about the long-term impact in regard to the training of professionals. Future research could expand knowledge on the HPIPS development by (1) focusing on interprofessional and inter-universities boundaries interventions, (2) developing a national strategy in health promotion as a curricular program with early initiation, continuity of learning over time, and a progression of skill levels according to teaching cycles, (3) and set up evaluations of interventions at the national level in order to measure the impact of this program on the health of populations and on the health promotion skills of students.

## Data Availability

The datasets used and/or analyzed during this study are available from the corresponding author upon reasonable request.

## Abbreviations

ARS: Regional Health Agency (Agence Régionale de Santé)
CODES: Departmental Health Education Committee (Comité Départemental d’Éducation pour la Santé)
CER: Research Ethics Committee (Comité d’Éthique de la Recherche)
ED: Supervised teaching (Enseignements Dirigés)
HCSP: French High Council for Public Health (Haut Conseil de la Santé Publique)
IFMK: Physiotherapy training institute (Institut de Formation en Masso-Kinésithérapie)
IFSI: Nursing Care Training Institute (Institut de Formation en Soins Infirmiers)
INSPÉ: French National Institute for Higher Education (Institut National Supérieur du Professorat et de l’Éducation)
IREPS: Regional health education and promotion body (Instance Régionale d’Education et de Promotion Santé)
UR: Research unit
WHO: World Health Organization
HPIPS: National Health Promotion Intervention Program (Service Sanitaire des Étudiants en Santé)
UFR: Training and Research Units (Unités de Formation et de Recherche)
